# Automated Individual-Level ROI-to-Spectrum Extraction for Hyperspectral Analysis in Forensic Entomology

**DOI:** 10.3390/insects17080757

**Published:** 2026-07-23

**Authors:** Yang Xia, Hai Wu, Hao Wang, Guojing Xu, Changbo Chen, Fuxin Song, Yihong Qu, Xiangyan Zhang

**Affiliations:** Department of Forensic Science, Xiangya School of Basic Medical Sciences, Central South University, Changsha 410013, China; csu_xiayang@163.com (Y.X.); wuhai@csu.edu.cn (H.W.);

**Keywords:** forensic entomology, hyperspectral imaging, automated ROI extraction, spectral preprocessing, individual-level spectra

## Abstract

Hyperspectral imaging (HSI) has potential for forensic entomology, but its practical use is limited by manual region-of-interest (ROI) delineation before spectral extraction. This step is time-consuming, operator-dependent, and difficult to standardize across insect species and developmental stages. Here, we developed an automated individual-level ROI-to-spectrum workflow for HSI analysis of forensically important insects. The dataset included 63 hyperspectral images and 1868 manually annotated insect individuals, covering larvae, pupae, and adults. The proposed Hyperspectral Imaging Fully Convolutional Network (HSI-FCN) segmented insect body regions from three-band pseudo-RGB images, back-projected the predicted masks to the original HSI data cubes, generated individual-level ROIs, and extracted full-band mean spectra. On an independent test set containing 204 insect individuals, HSI-FCN achieved mean Dice and intersection over union (IoU) values of 0.9079 and 0.8328, respectively, and showed the best overall performance among representative segmentation models. All test individuals were successfully matched with their corresponding manual ROIs. Spectra extracted from automated ROIs were highly consistent with manual ROI spectra, with a mean spectral angle mapper of 3.06° and a Pearson correlation coefficient of 0.9956. These results show that the proposed workflow can replace manual ROI delineation with a reproducible preprocessing step for insect HSI analysis, supporting standardized spectral extraction and future applications in forensic entomology.

## Simple Summary

Hyperspectral imaging (HSI) is useful in forensic entomology because it records both image and spectral information from insect samples. However, current insect HSI studies often require manual drawing of regions of interest (ROIs) before spectral extraction, which is slow and difficult to standardize. In this study, we developed an automated workflow to segment insect bodies and extract individual-level spectra from hyperspectral images. The method was tested on larvae, pupae, and adults of forensically important insects. It successfully matched all insect individuals in the independent test set with manual annotations and produced spectra highly similar to those obtained from manual ROIs. This workflow can reduce manual work and improve the reproducibility of insect hyperspectral analysis.

## 1. Introduction

Hyperspectral imaging (HSI) is an optical detection technique that integrates two-dimensional imaging with continuous spectral acquisition, enabling simultaneous recording of the spatial distribution of a sample and its narrow-band reflectance spectra within a single data cube [1]. Compared with conventional RGB imaging, HSI not only captures morphological, color, and surface structural features of the target but also provides spectral information related to material composition, tissue status, and optical properties [2]. HSI is non-contact and non-destructive, provides high-dimensional information, and enables spatially localized analysis. It has therefore been widely applied in food quality and safety assessment [3,4], crop phenotyping [5,6], medical tissue analysis [7,8,9,10], and forensic trace detection [11]. These applications indicate that HSI is particularly suitable for complex sample analysis tasks requiring the simultaneous acquisition of spatial localization and spectral features.

Forensic entomology provides scientific evidence for forensic issues such as time since death, place of death, and corpse relocation by studying the community succession and development of necrophagous insects [12]. In real forensic cases, larvae, pupae, or adults of necrophagous insects are commonly used to estimate the minimum postmortem interval (PMImin). In recent years, HSI has gradually been introduced into forensic entomology as a non-destructive detection technique. During insect development, biological processes such as pigment deposition, cuticular sclerotization, tissue remodeling, and changes in metabolic status can induce continuous changes in reflectance spectra [13]. Previous studies have shown that reflectance spectra obtained from insect puparia or body regions can be used to distinguish species and developmental stages [14,15]. When combined with machine learning models, HSI can further be used for pupal age estimation in important necrophagous insects such as *Chrysomya megacephala* (Fabricius, 1794) (Diptera: Calliphoridae) [13], *Sarcophaga formosensis* (Kirner & Lopes, 1961) (Diptera: Sarcophagidae) [16], and *Dermestes maculatus* DeGeer, 1774 (Coleoptera: Dermestidae) [17]. These studies demonstrate the potential of HSI for non-destructive analysis of forensically important insects.

However, current HSI studies in forensic entomology still face clear limitations in data preprocessing. Most studies rely on manual region-of-interest (ROI) delineation using software such as ENVI, followed by extraction of individual spectra based on the mean reflectance of all pixels within each ROI [13,16,17]. This workflow is feasible in small-scale exploratory studies, but it is difficult to meet the demands of high-throughput analysis under large-sample, multi-species, multi-stage, and multi-batch imaging conditions. Manual ROI delineation is not only time-consuming and labor-intensive but is also susceptible to operator experience, annotation criteria, and fatigue, thereby reducing the reproducibility of data processing [18,19]. More importantly, target boundaries, insect posture, shadowed regions, and background noise in hyperspectral images can all affect ROI annotation quality [20]. Downstream spectral modeling usually relies on the mean spectrum of pixels within an ROI. Therefore, even a small amount of background pixel inclusion or boundary omission may alter individual spectral features and further affect the stability of age estimation, species identification, or developmental status classification models.

Automatic image segmentation provides a feasible solution to the low efficiency and insufficient standardization of manual ROI delineation [21]. Automated ROI extraction can convert batch hyperspectral images into standardized specimen-level ROIs and full-band reflectance spectra with reduced operator intervention in forensic entomology HSI workflows. These spectra can directly support downstream tasks such as species identification, developmental-stage determination, and insect developmental age estimation for PMImin inference. In medical imaging [22,23], remote sensing monitoring [24], and other HSI application scenarios, deep learning-based segmentation methods have been used for lesion recognition, land-cover classification, and hyperspectral semantic segmentation, showing potential for improving processing efficiency and reducing human subjectivity. Similar automated ROI strategies have been applied in insect-related near-infrared hyperspectral imaging (NIR-HSI) analysis, such as identifying puncture-damage regions caused by *Halyomorpha halys* (Stål, 1855) (Hemiptera: Pentatomidae) on pears, demonstrating their value for objective and task-specific spectral extraction [19]. However, insect HSI images have characteristics that differ from those of medical tissues and remote sensing scenes. Insect samples are usually small in scale, densely distributed, and variable in posture, with complex backgrounds and shadow conditions; moreover, spectral extraction is highly sensitive to ROI purity. Therefore, existing HSI segmentation methods cannot directly demonstrate their suitability for individual-level ROI generation in insect hyperspectral images. The evaluation of automatic segmentation should not be limited to spatial overlap metrics such as Dice or intersection over union (IoU). It should also verify whether automatically generated ROIs can extract full-band reflectance spectra consistent with those obtained from manual ROIs.

To address these issues, this study constructed an insect hyperspectral image dataset containing larval, pupal, and adult samples, covering different insect taxa, developmental stages, and imaging backgrounds. We further proposed a Hyperspectral Imaging Fully Convolutional Network (HSI-FCN) for automatic insect body segmentation. The method takes three-band pseudo-RGB images as input, automatically generates insect body masks, back-projects them to the original HSI data cube to construct individual-level ROIs, and extracts full-band mean spectra. In addition, we compared HSI-FCN with LightSegCNN, U-Net, U-Net+SimCLR, and YOLOv8n-seg and evaluated its performance in terms of pixel-level segmentation, individual-level ROI localization, and spectral fidelity. This study aims to provide an objective, standardized, and reproducible preprocessing workflow for forensic entomology hyperspectral data analysis and to establish a methodological basis for subsequent HSI-based insect species identification, developmental stage discrimination, and PMImin-related modeling.

## 2. Materials and Methods

### 2.1. Hyperspectral Image Dataset and Manual Annotations

In this study, we constructed a dataset for automatic insect hyperspectral image segmentation and individual-level spectral extraction. The dataset contained 63 hyperspectral images from six subsets, namely SF20, SF25, SF30, SP25, AD, and LA, comprising a total of 1868 manually annotated insect individuals. The SF20, SF25, and SF30 subsets were derived from raw HSI images and manual ROI annotations from a previous pupal development study of *S. formosensis* [16]. These data were repurposed in the present study from developmental age estimation to automatic ROI segmentation and individual-level full-band spectral extraction. The SP25 subset was derived from pupae of *Sarcophaga peregrina* (Robineau-Desvoidy, 1830) (Diptera: Sarcophagidae). The AD subset included adults of five necrophagous fly species: *C. megacephala*, *Chrysomya rufifacies* (Macquart, 1842) (Diptera: Calliphoridae), *Chrysomya pinguis* (Walker, 1858) (Diptera: Calliphoridae), *Chrysomya nigripes* Aubertin, 1932 (Diptera: Calliphoridae), and *Lucilia sericata* (Meigen, 1826) (Diptera: Calliphoridae). The LA subset included larvae of two fly species, *C. megacephala* and *L. sericata*. By including different insect species and developmental stages, this dataset was used to further evaluate the applicability of the model under different imaging backgrounds and sample morphologies. All HSI data cubes were acquired using the same Snapscan visible and near-infrared (VNIR) hyperspectral imaging system (imec, Leuven, Belgium) and were radiometrically corrected using white and dark references. The number of effective spectral bands ranged from 128 to 150. The SF20, SF25, SF30, SP25, and AD subsets contained 150 effective bands, whereas the LA subset contained 128 effective bands.

Manual ROIs were annotated using ENVI 5.6 (Harris Geospatial Solutions, Inc., Broomfield, CO, USA), with each ROI corresponding to one individual insect. These ROIs served as the reference standard for model training and evaluation. All ROI files were parsed and converted into binary masks aligned with the spatial coordinates of the original HSI images. Pixels inside the ROIs were labeled as insect body regions, whereas pixels outside the ROIs were labeled as background. The converted masks were uniformly inspected to exclude obvious coordinate offsets, missing annotations, duplicate annotations, or format conversion errors.

The dataset was stratified by subset and randomly divided into training, validation, and test sets containing 48, 8, and 7 images, respectively. The test set was kept independent during model training and parameter optimization and was used only for final evaluation. The independent test set contained seven images covering all six subsets, with a total of 204 ground truth (GT) annotated individuals. The number of images, manually annotated individuals, independent test images, and annotated individuals in the test images for each subset are shown in Table 1.

### 2.2. Construction of Pseudo-RGB Images and Preprocessing

Band 120, Band 80, and Band 40 were selected from each HSI data cube and mapped to the red (R), green (G), and blue (B) channels of a pseudo-RGB image, respectively, to reduce HSI dimensionality and adapt the data to a two-dimensional convolutional network. These bands were within the shared spectral range of all subsets, including the LA subset with 128 bands, and were approximately evenly distributed along the spectral axis. This band combination covers the near-infrared, red-edge, and visible regions, providing broad spectral coverage and main spatial contrast between insect bodies and the background, although it was not derived from exhaustive band optimization. Each channel was then normalized using min–max normalization and uniformly resized to 256 × 256 pixels as the model input. The corresponding binary masks were processed using the same cropping window, scaling ratio, and coordinate transformation parameters to ensure pixel-level correspondence between images and labels. Area interpolation was used for image resizing, and nearest-neighbor interpolation was used for mask resizing to preserve the binary nature of the labels.

During preprocessing, the cropping window, scaling ratio, and coordinate offset parameters were recorded for each image. After model prediction, the predicted masks at 256 × 256 resolution were back-projected to the original HSI image coordinate system using these parameters for subsequent individual-level ROI generation and full-band spectral extraction.

### 2.3. Network Architecture of the Proposed HSI-FCN

**Figure 1:**
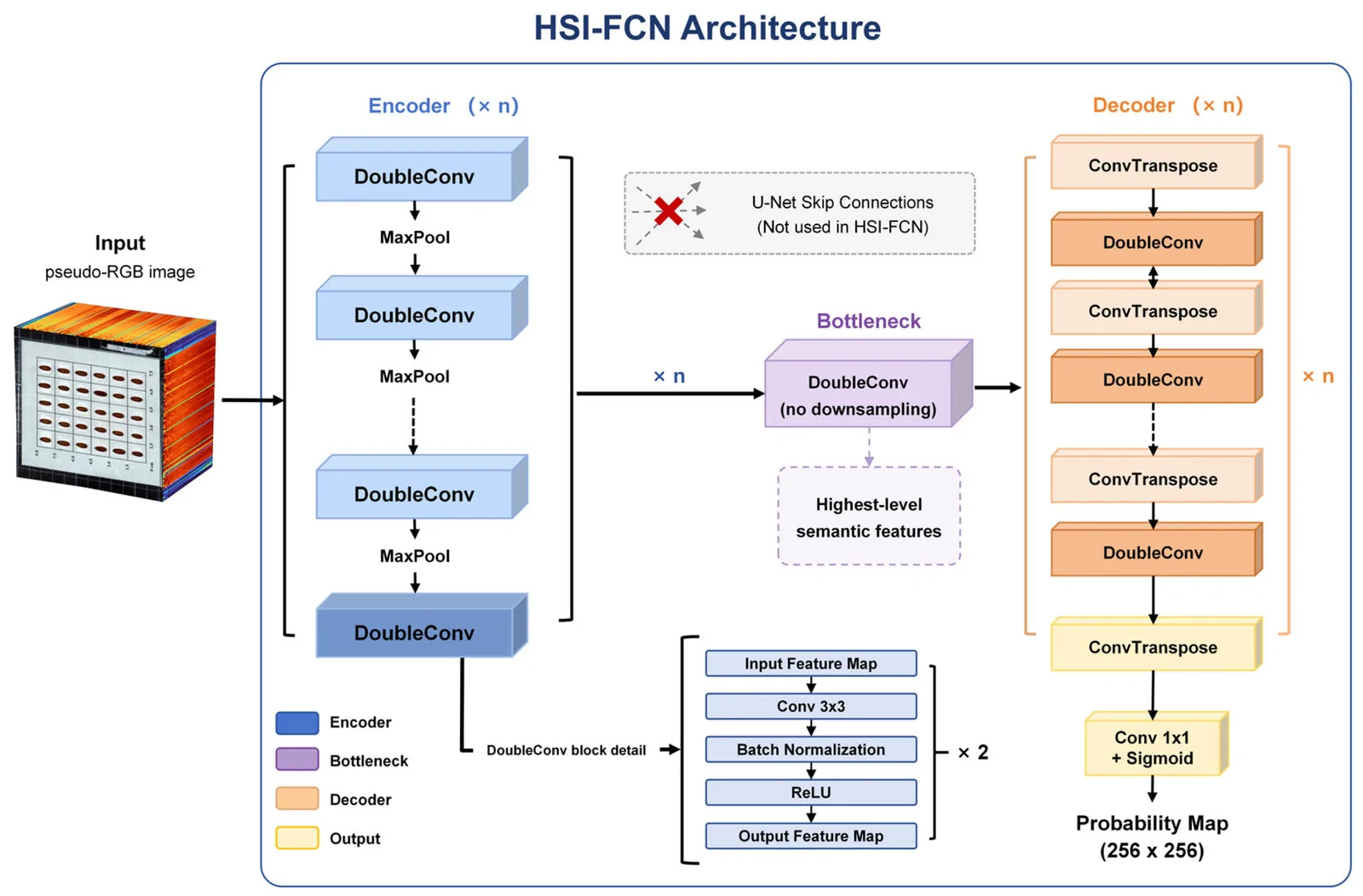
Architecture of the proposed HSI-FCN for insect body segmentation. The network takes a pseudo-RGB image as input and generates a single-channel probability map with the same spatial size. HSI-FCN consists of an encoder, a bottleneck layer and a decoder. Each DoubleConv block contains two consecutive 3 × 3 convolutional layers, each followed by batch normalization and ReLU activation. Solid black arrows indicate forward feature propagation through the network. Gray dashed arrows crossed by a red × indicate the U-Net skip connections that are not used in HSI-FCN. Blue, purple, orange, and yellow blocks represent the encoder, bottleneck, decoder, and output modules, respectively.

We constructed a fully convolutional semantic segmentation network, named HSI-FCN. It was designed for pixel-level segmentation of pseudo-RGB images generated from insect HSI data. HSI-FCN uses an encoder–decoder architecture with four downsampling levels, a central bottleneck layer and four upsampling levels. The overall framework is shown in Figure 1. The encoder extracts multi-scale contextual features from the input image. The decoder gradually restores spatial resolution through transposed convolution and outputs a pixel-level probability map for the insect body region.

Unlike the standard U-Net, HSI-FCN does not use skip connections between the encoder and the corresponding decoder layers. Each decoder layer receives only the upsampled feature map from the previous layer and does not concatenate shallow high-resolution features from the encoder. This design reduces the direct influence of shallow texture and background details on the decoder, while also decreasing the complexity of model parameters and feature fusion, making it suitable for small-sample insect hyperspectral image segmentation.

The model input was a three-channel pseudo-RGB image of 256 × 256 pixels, and the output was a single-channel probability map of the same size. Each downsampling level in the encoder consisted of a double convolution module and a 2 × 2 max-pooling operation. The double convolution module contained two consecutive 3 × 3 convolutional layers, each followed by batch normalization and a rectified linear unit (ReLU) activation function. The base number of channels was set to 48, and the encoder channel numbers were 48, 96, 192, and 384, respectively. The central bottleneck layer contained 768 channels.

The decoder restored spatial resolution step by step using transposed convolutions and further refined the upsampled features through double convolution modules. The decoder channel numbers were gradually reduced from 768 to 384, 192, 96, and 48. To reduce the risk of overfitting, Dropout2D regularization was applied to the intermediate decoder layers, with a dropout rate of 0.15. Figure 1 illustrates the overall encoder–bottleneck–decoder structure of HSI-FCN, the composition of the DoubleConv modules, and the difference from U-Net skip connections. Finally, a 1 × 1 convolution mapped the 48-channel feature map to a single-channel output, and a Sigmoid function was used to obtain the predicted probability of each pixel belonging to the insect body region.

### 2.4. Loss Function and Optimization

Considering the class imbalance between insect body and background regions, the model was optimized using a joint loss function, $L_{\mathrm{Total}}$, consisting of Weighted Binary Cross-Entropy Loss ($L_{\mathrm{BCE}}$) and Dice loss based on the Dice similarity coefficient. The total loss was defined as:

$$L_{\mathrm{Total}}\;=\;L_{\mathrm{BCE}}\;+\;L_{\mathrm{Dice}}$$

Conventional cross-entropy loss can be dominated by many simple background pixels. By introducing a positive-sample weighting factor ($w_{p}$), the model increases the penalty for misclassifying the minority class, namely insect-body pixels. The calculation was as follows:

$$L_{\mathrm{BCE}}\;=-\frac{1}{N}\sum_{i=1}^{N}w_{p}\cdot y_{i}\cdot \log{(p}_{i})\;+\left(1-y_{i}\right)\cdot \log{(1-p}_{i})$$

where *N* denotes the total number of pixels in the image; $y_{i}\;\in \;\{0,\;1\}$ is the ground truth label of the *i*-th pixel, with 1 representing insect body pixels and 0 representing background pixels; $p_{i}$ is the probability predicted by the network after Sigmoid activation that the *i*-th pixel belongs to the insect body region; and $w_{p}$ was set to 12 to balance the class distribution.

The Dice loss was defined as:

$$L_{\mathrm{Dice}}\;=\;1-\frac{2\sum_{i=1}^{N}\left(p_{i}y_{i}\right)\;+\;\epsilon }{\sum_{i=1}^{N}\left(y_{i}\right)\;+\sum_{i=1}^{N}\left(p_{i}\right)\;+\;\epsilon }$$

where $\sum_{i=1}^{N}\left(p_{i}y_{i}\right)$ represents the intersection between the predicted probability map and the ground truth binary mask. To avoid division by zero and maintain numerical stability during the early stage of training, a smoothing term ϵ was added to both the numerator and denominator; in this study, ϵ was set to 1 × 10^−6^.

$L_{\mathrm{BCE}}$ emphasizes pixel-level classification accuracy, whereas $L_{\mathrm{Dice}}$ emphasizes the overall spatial overlap between the predicted mask and the reference mask. Combining these two losses helps achieve more stable segmentation results under class-imbalanced conditions.

### 2.5. Model Training, Inference and Post-Processing

Data augmentation was applied to the training set to increase sample diversity and reduce overfitting risk under small-sample conditions. Eight augmented samples were generated from each training image. Thus, the number of training samples per epoch increased from 48 original images to 384 samples. The augmentation operations included random horizontal flipping, random vertical flipping, random rotation, random scaling, brightness perturbation and Gaussian noise perturbation. The random scaling range was 0.85–1.15. The flipping probability was 0.5. The rotation angles were 0, 90, 180 and 270 degrees. The perturbation amplitude was ±15%, and the Gaussian noise standard deviation was *σ* = 0.03.

Model training used a batch size of 2 and a maximum of 200 epochs. The validation Dice coefficient was used as the model-selection metric. The model weights with the highest validation Dice were saved. An early-stopping strategy was used to avoid late-stage overfitting. Training was stopped when validation Dice did not improve for 40 consecutive epochs.

The AdamW optimization algorithm was used for parameter updating, with an initial learning rate of 3 × 10^−4^ and a weight decay coefficient of 2 × 10^−4^. The learning rate was scheduled using the CosineAnnealingWarmRestarts strategy, with an initial period of 20, a period multiplication factor of 2, and a minimum learning rate of 1 × 10^−6^. All random processes were controlled using a fixed random seed, with SEED set to 2024.

During inference, test-time augmentation (TTA) was used. Eight geometric transformations were applied to each test image, including the original image, horizontal flipping, vertical flipping, combined horizontal and vertical flipping, 90° rotation, 180° rotation, 270° rotation, and vertical flipping followed by 90° rotation. Each transformed image was predicted by the model, and the resulting probability maps were inversely transformed back to the original orientation. The eight probability maps were then averaged pixel by pixel to obtain the final prediction probability map. The final probability map was binarized using a threshold of 0.5 to generate an initial predicted mask. A 3 × 3 elliptical structuring element was then used for one morphological opening operation and one morphological closing operation to remove isolated noise points and fill small holes. The post-processed mask was further analyzed using 8-connectivity connected-component analysis, and connected regions with an area smaller than 0.05% of the total image pixels were removed.

The post-processed 256 × 256 binary mask was back-projected to the original HSI image coordinate system using nearest-neighbor interpolation. Individual-level ROIs were then generated based on the connected-component results. The reflectance values of all pixels across the full spectral range were extracted from each ROI, and the mean spectrum was calculated as the spectral feature of that individual. In addition, the automatically generated ROI coordinates were exported in an ENVI-compatible format for subsequent manual inspection and spectral analysis.

### 2.6. Evaluation Metrics

The model was evaluated at three levels: pixel-level segmentation accuracy, individual-level ROI localization, and spectral fidelity.

Pixel-level evaluation was performed in the original HSI image coordinate system. The back-projected predicted masks were compared pixel by pixel with the corresponding GT masks, and Dice, IoU, Precision, Recall, and Accuracy were calculated. Dice was used as the primary segmentation metric to measure the overall spatial overlap between the predicted mask and the reference mask and was defined as:

$$\mathrm{Dice}\;=\;\frac{(2\;\times \;\mathrm{TP})}{(2\;\times \;\mathrm{TP}\;+\;\mathrm{FP}\;+\;\mathrm{FN}\;+\;\epsilon )}$$

where TP, FP, and FN represent true-positive, false-positive, and false-negative pixels, respectively. IoU, Precision, Recall, and Accuracy were used as complementary metrics to evaluate region overlap, prediction purity, target completeness, and overall classification accuracy.

Individual-level ROI localization was evaluated based on connected-component analysis. After 8-connectivity analysis was performed on both the predicted mask and the GT mask, each connected component was regarded as an individual region. A predicted region was considered successfully matched to a GT region when the Polygon IoU between them was ≥0.1. Based on this rule, the numbers of matched individuals, missed regions, and false-positive regions were calculated. Polygon IoU, area ratio, and Hausdorff distance were further calculated for successfully matched individuals to evaluate the geometric consistency between automatically generated ROIs and manual ROIs.

Spectral fidelity was evaluated based on the original HSI data cube. Full-band pixel reflectance values were extracted from both the predicted ROI and the corresponding GT ROI for each successfully matched individual, and mean spectra were calculated. Differences between the mean spectra extracted from predicted and GT ROIs were evaluated using the Spectral Angle Mapper (SAM), Pearson correlation coefficient, coefficient of determination (*R*^2^), and root mean square error (RMSE). SAM was used to measure the angular difference between two spectral curves in high-dimensional spectral space and was defined as:

$$\mathrm{SAM}\;=\;\arccos\left(\frac{\sum_{j=1}^{B}G_{j}P_{J}}{\sqrt{\sum_{J=1}^{B}G_{j}^{2}}\sqrt{\sum_{J=1}^{B}P_{j}^{2}}}\right)\;\times \;\frac{180}{\pi }$$

where *G_j_* and *P_j_* denote the reflectance values of the mean spectra extracted from the GT ROI and the predicted ROI at the *j*-th band, respectively, and B denotes the number of spectral bands. The Pearson correlation coefficient, *R*^2^, and RMSE were used to evaluate the linear correlation, fitting consistency, and absolute error between the two mean spectra, respectively.

## 3. Results

### 3.1. Pixel-Level Segmentation Performance of HSI-FCN on the Independent Test Set

The pixel-level segmentation performance of HSI-FCN for insect body regions was evaluated using the independent test set. The test set contained seven hyperspectral images covering the six subsets AD, LA, SF20, SF25, SF30, and SP25, with a total of 204 GT-annotated insect individuals. The 256 × 256 binary masks predicted by the model were back-projected to the original HSI image coordinates and then compared pixel by pixel with the corresponding GT masks. Therefore, all pixel-level metrics were calculated at the original image resolution, and the complete image-wise results are provided in Table S1.

Across the seven test images, HSI-FCN achieved mean Dice, IoU, Precision, Recall, and Accuracy values of 0.9079 ± 0.0332, 0.8328 ± 0.0542, 0.9012 ± 0.0622, 0.9188 ± 0.0478, and 0.9898 ± 0.0034, respectively. The image-wise Dice values ranged from 0.8472 to 0.9382, and the IoU values ranged from 0.7350 to 0.8836, indicating moderate performance variation among the test images. SP25-2 achieved the highest Dice and IoU values, at 0.9382 and 0.8836, respectively, followed by SF30-7, with Dice and IoU values of 0.9302 and 0.8695, respectively. The remaining SF test images also maintained high Dice values, with 0.9226 for SF20-11, 0.9205 for SF20-4, and 0.9204 for SF25-9, indicating that HSI-FCN achieved high region-overlap accuracy in the SF- and SP-related images.

The image-wise Precision and Recall results showed different error patterns across test images. SF30-7 and SF20-4 showed high Precision values of 0.9750 and 0.9500, respectively, indicating a low proportion of background pixels in the predicted foreground regions. SP25-2, SF25-9, and SF20-11 showed Recall values of 0.9670, 0.9622, and 0.9557, respectively, indicating that most GT insect body pixels in these images were covered by the predicted regions. In contrast, AD_CMH-1 had the lowest Recall value among all test images, at 0.8380, suggesting relatively more missed insect body pixels. LA_LSLF-2 had the lowest Precision value, at 0.7806, indicating relatively more background pixel misclassification.

**Figure 2:**
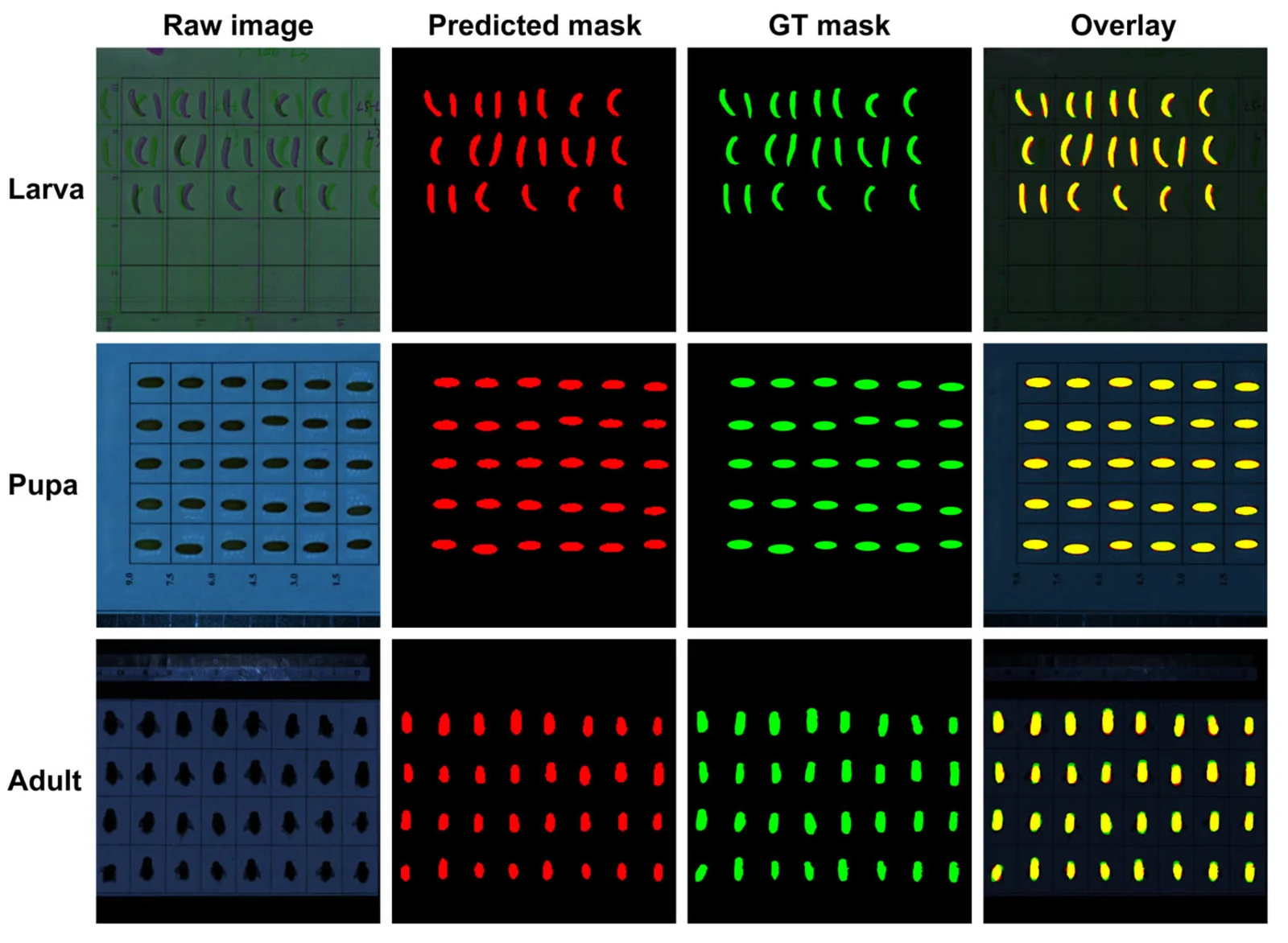
Representative segmentation results of HSI-FCN for larval, pupal and adult insect samples. Each row represents one insect developmental stage, and the columns show the raw pseudo-RGB image, HSI-FCN-predicted mask, GT mask, and overlay result. In the overlay images, yellow regions indicate overlapping pixels between the predicted and GT masks, red regions indicate prediction-only pixels, and green regions indicate GT-only pixels.

Figure 2 shows representative raw pseudo-RGB images, HSI-FCN-predicted masks, GT masks, and overlay results for larval, pupal, and adult test samples. Across all three developmental stages, the predicted regions covered the main GT insect body regions and showed high spatial overlap in the overlay images. The predicted masks of pupal samples were highly consistent with the GT masks, whereas local boundary deviations and minor missed or false-positive regions were still observed in larval and adult samples. These visual results were consistent with the quantitative results in Table S1. HSI-FCN achieved Dice values above 0.84 for all test images and maintained a mean Recall of 0.9188 while achieving a mean Precision of 0.9012, indicating that the model can provide a stable pixel-level segmentation basis for individual-level ROI construction and full-band spectral extraction under different insect morphologies.

### 3.2. Comparison of HSI-FCN with Representative Segmentation Models

**Figure 3:**
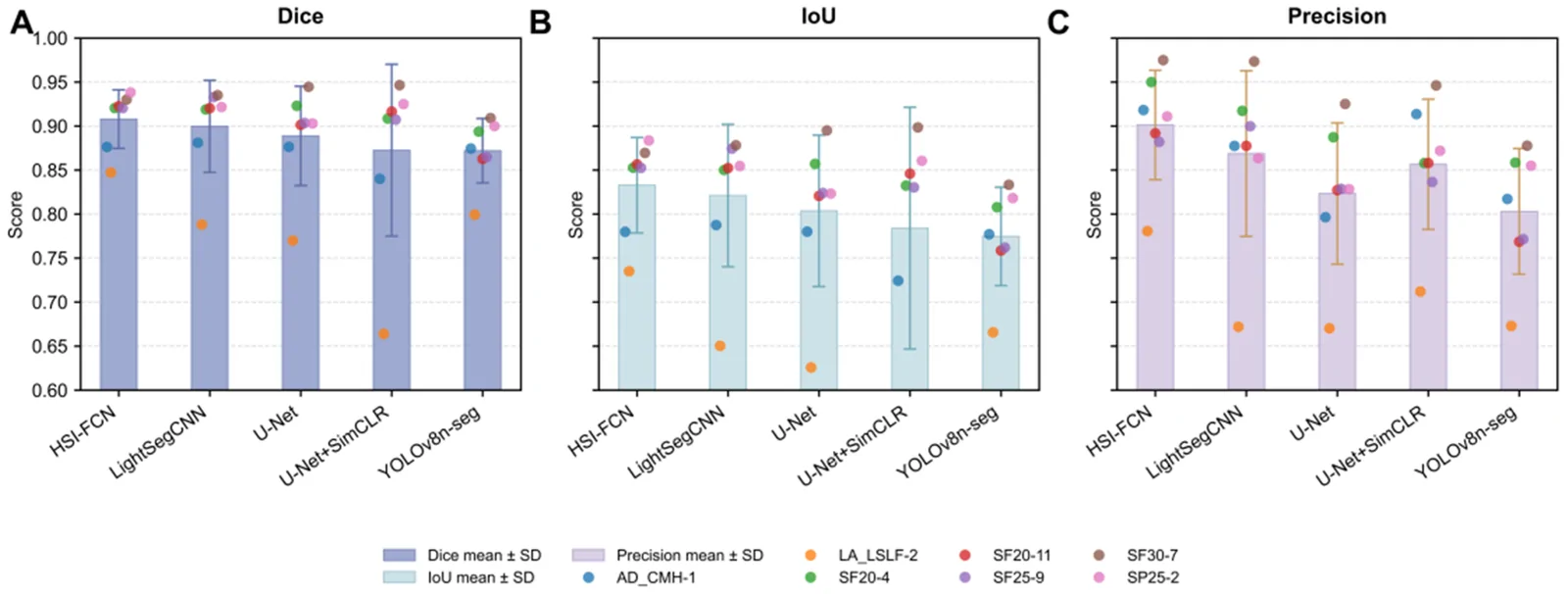
Comparison of segmentation performance among five representative models. (**A**) Dice; (**B)** IoU; and (**C**) Precision. Bars represent the mean values across the seven independent test images for HSI-FCN, LightSegCNN, U-Net, U-Net+SimCLR, and YOLOv8n-seg. Error bars indicate standard deviations, and overlaid points represent individual test images.

To further evaluate the segmentation performance of HSI-FCN, four representative models were selected for comparison: LightSegCNN, U-Net, U-Net+SimCLR, and YOLOv8n-seg. All models were evaluated on the same independent test set using a consistent back-projection and original-resolution evaluation procedure. The mean segmentation performance of the five models is shown in Table 2, and visual comparisons of Dice, IoU, and Precision are shown in Figure 3.

HSI-FCN achieved the best overall segmentation performance among the five representative models, ranking highest in Dice, IoU, Precision, and Accuracy (Figure 3, Table 2). Its advantage in Precision was particularly evident, indicating that HSI-FCN introduced the lowest proportion of background pixels into the predicted insect body regions and achieved the best overall region-overlap accuracy. In contrast, although U-Net and YOLOv8n-seg showed higher Recall values, their Precision values were markedly lower, suggesting that these two models tended to enlarge the predicted foreground regions. This allowed them to cover more GT insect body pixels but also introduced more background false positives. Although HSI-FCN did not achieve the highest Recall, it provided a better balance between target coverage and prediction purity. LightSegCNN showed overall performance close to that of HSI-FCN, but its Dice, IoU, and Precision remained slightly lower. U-Net+SimCLR achieved higher Precision than U-Net and YOLOv8n-seg, but its overall segmentation performance still did not exceed that of HSI-FCN, indicating that pretraining enhancement did not improve comprehensive performance on the current test set. Overall, the comparison among the five models shows that HSI-FCN substantially improved prediction purity while maintaining high Recall, making it more suitable for subsequent automatic ROI-based full-band spectral extraction. The independent test set contained only seven HSI images; therefore, this model comparison was treated as a descriptive evaluation rather than a formal inferential statistical test. Nevertheless, all models were evaluated on the same test images using identical post-processing, back-projection, and metric-calculation procedures.

### 3.3. Individual-Level ROI Localization Performance

The pixel-level segmentation results provided the basis for further analysis of the stability of converting HSI-FCN-predicted masks into individual-level ROIs. Connected-component analysis showed that the GT masks of the seven independent test images contained 204 insect body regions in total, and HSI-FCN also generated 204 predicted regions. All predicted regions were successfully matched to their corresponding GT regions. Therefore, the individual-level ROI matching rate reached 100%, with no missed or extra false-positive regions (Table 3).

Overall, the mean Polygon IoU of the 204 matched ROI pairs was 0.836 ± 0.059, indicating high spatial consistency between the automatically generated ROIs and the manually annotated ROIs. The mean area ratio between predicted ROIs and GT ROIs was 1.019 ± 0.116, which was close to 1, suggesting that the model showed no obvious systematic over-expansion or contraction. The mean Hausdorff distance was 37.7 ± 10.6 px, indicating that some local boundary differences remained between automatic and GT ROIs, although the overall target localization was consistent.

Geometric consistency varied among the test images. SP25-2 achieved the highest Polygon IoU, at 0.883 ± 0.023. SF30-7, SF20-11, SF25-9, and SF20-4 also maintained high Polygon IoU values of 0.869 ± 0.021, 0.857 ± 0.033, 0.854 ± 0.046, and 0.853 ± 0.024, respectively. In contrast, AD_CMH-1 and LA_LSLF-2 showed lower Polygon IoU values of 0.777 ± 0.043 and 0.733 ± 0.042, respectively, indicating relatively weaker boundary consistency between predicted ROIs and GT ROIs.

The area ratio further reflected the direction of segmentation errors in different test images. AD_CMH-1 and SF30-7 had area ratios of 0.912 ± 0.081 and 0.913 ± 0.035, respectively, indicating that the predicted ROIs were slightly smaller than the GT ROIs. In contrast, LA_LSLF-2 had an area ratio of 1.191 ± 0.105, suggesting a tendency toward ROI over-expansion. This was consistent with the lower Precision observed for LA_LSLF-2 in Section 3.1, indicating that its error mainly resulted from the inclusion of background regions in the predicted insect body areas. Overall, HSI-FCN stably generated individual-level ROIs corresponding one-to-one with manually annotated regions, providing a reliable geometric basis for subsequent individual-level full-band spectral extraction.

### 3.4. Spectral Fidelity of Spectra Extracted from Automatically Generated ROIs

We then compared the consistency between full-band spectra extracted from automatically generated ROIs and those extracted from GT ROIs after confirming stable individual-level ROI generation by HSI-FCN. This analysis was based on 204 matched insect individuals from the seven test images, and SAM, Pearson *r*, *R*^2^, and RMSE were calculated. All spectra were extracted from the back-projected ROI regions in the original HSI data cubes rather than from the three-band pseudo-RGB input images, allowing the actual effect of automatic ROIs on full-band spectral extraction to be evaluated.

**Figure 4:**
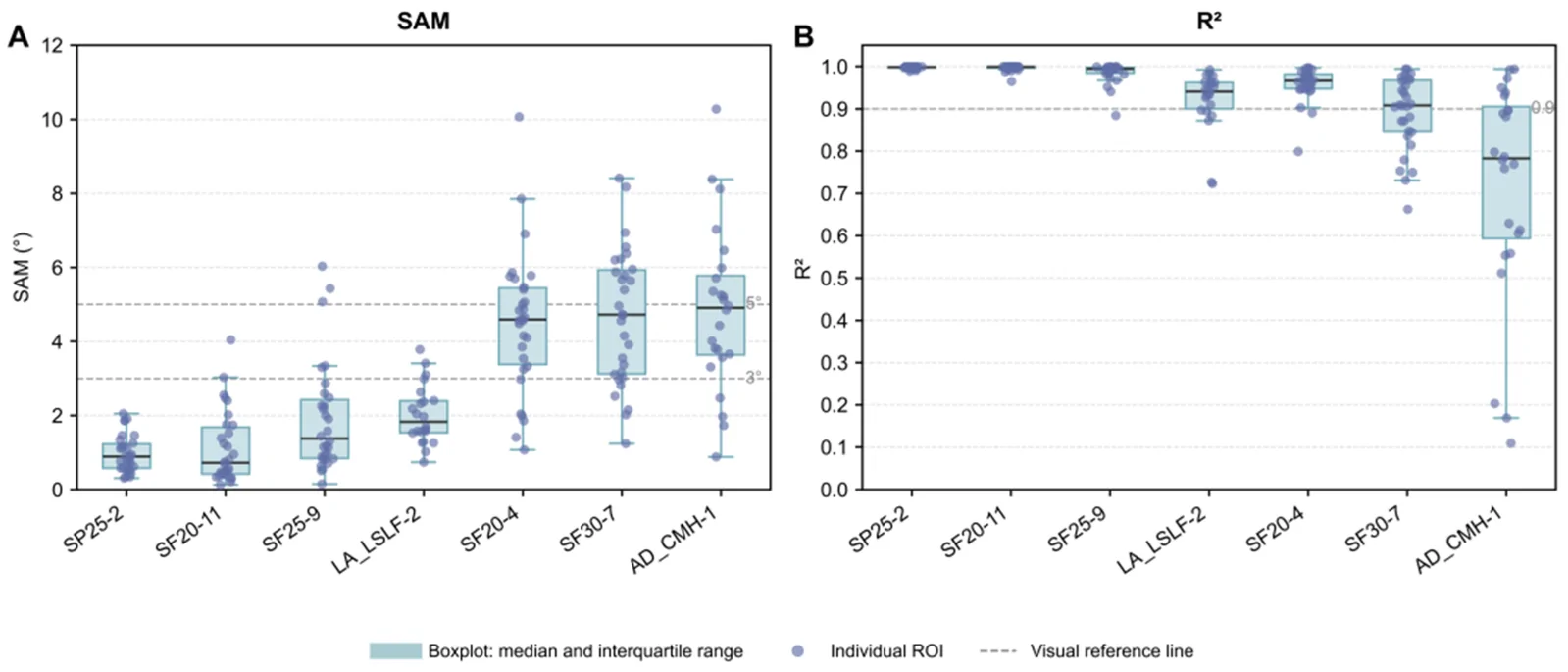
Distribution of spectral fidelity metrics for HSI-FCN-derived ROIs across the seven test images. Boxplots show the distributions of SAM (**A**) and *R*^2^ (**B**) calculated from matched individual ROIs. Each point represents one individual insect body ROI. Test images are ordered by increasing mean SAM. Dashed lines indicate visual reference thresholds. In the visualization, AD_CMH-1 individuals with negative *R*^2^ values were excluded from both SAM and *R*^2^ boxplots.

Overall, spectra extracted from HSI-FCN-derived ROIs showed good agreement with those extracted from GT ROIs in terms of spectral curve shape, although different spectral fidelity metrics reflected different aspects of error (Table 4, Figure 4). Across the 204 individuals, the mean SAM was 3.06 ± 2.45°, Pearson *r* was 0.9956 ± 0.0123, *R*^2^ was 0.848 ± 0.467, and RMSE was 0.0132 ± 0.0103. The SAM distribution showed that 118 of 204 individuals (57.8%) had SAM values below 3°, 158 of 204 individuals (77.5%) had SAM values below 5°, and only 3 of 204 individuals (1.5%) had SAM values above 10°. The low SAM values and high Pearson *r* values jointly indicate that the automatic ROIs generally preserved the main spectral curve shapes and inter-band variation patterns of the GT ROIs. In contrast, *R*^2^ and RMSE were more sensitive to reflectance amplitude shifts and local band-wise errors, and their larger standard deviations suggest that some individuals still showed absolute reflectance differences between automatic and GT ROIs despite similar spectral shapes. Therefore, in this study, SAM and Pearson *r* were mainly used to evaluate spectral shape fidelity, whereas *R*^2^ and RMSE were interpreted as complementary indicators of amplitude consistency and absolute error.

Spectral fidelity varied among the test images (Table 4, Figure 4). SP25-2, SF20-11, and SF25-9 showed the best performance, with mean SAM values of 0.97 ± 0.50°, 1.13 ± 0.98°, and 1.91 ± 1.49°, respectively, and corresponding *R*^2^ values of 0.998 ± 0.003, 0.997 ± 0.007, and 0.986 ± 0.024. These results indicate high consistency between spectra extracted from automatic and GT ROIs in both curve shape and reflectance amplitude. LA_LSLF-2 had a mean SAM of 2.02 ± 0.79°, but a relatively high RMSE of 0.0259 ± 0.0102, suggesting that the spectral shape was well preserved while some amplitude differences remained. SF20-4 and SF30-7 had SAM values of 4.50 ± 1.90° and 4.67 ± 1.83°, respectively, which were still mainly within a low spectral-angle range, indicating that the automatic ROIs generally retained the main spectral characteristics of the GT ROIs; however, their *R*^2^ and RMSE results suggested reflectance amplitude shifts in some individuals.

AD_CMH-1 was the main source of variation in spectral fidelity. This test image had the highest mean SAM value, at 5.80 ± 2.66°, and the lowest and most variable *R*^2^ value, at 0.244 ± 0.980. These results indicate that, for some individuals in this image, spectra extracted from automatic ROIs showed greater reflectance-amplitude differences from GT ROI spectra than those in the other test images, whereas the overall spectral curve morphology was still largely preserved, as reflected by the high Pearson *r* value. Combined with the pixel-level and ROI geometric evaluation results, AD_CMH-1 showed a relatively low Recall and an area ratio of 0.912 ± 0.081, suggesting that the predicted ROIs tended to be slightly smaller than the GT ROIs. This indicates that, in adult samples, local omission of insect body pixels or boundary differences may alter the pixel composition within ROIs and thereby affect the absolute reflectance level of the mean spectra. Therefore, although HSI-FCN-derived ROIs preserved good spectral shape fidelity for most test samples, amplitude consistency should still be interpreted cautiously using *R*^2^ and RMSE for boundary-sensitive samples.

**Figure 5:**
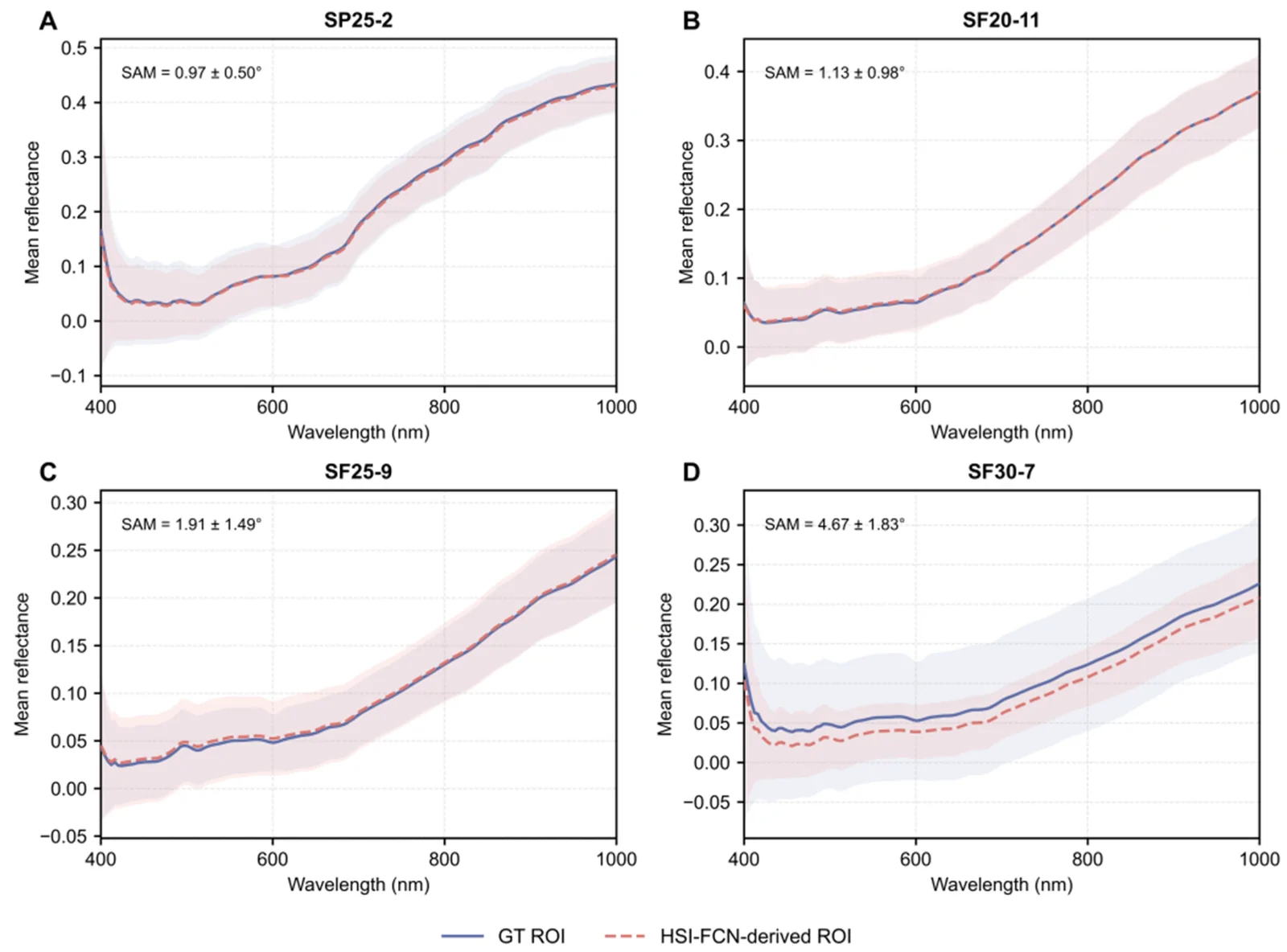
Mean spectra extracted from GT ROIs and HSI-FCN-derived ROIs in selected test images: (**A**) SP25-2; (**B**) SF20-11; (**C**) SF25-9; and (**D**) SF30-7. Mean reflectance spectra were calculated from GT ROIs and matched HSI-FCN-derived ROIs. Solid lines represent GT ROI spectra, and dashed lines represent spectra extracted from HSI-FCN-derived ROIs. Shaded areas indicate the standard deviation across individual ROIs.

Figure 5 shows the mean spectral curves extracted from GT ROIs and HSI-FCN-derived ROIs in representative test images, and the mean spectral curves for the remaining test images are shown in Figure S1. Overall, the mean spectral curves extracted from the two types of ROIs followed similar trends across the main spectral range, indicating that the automatic segmentation results preserved the major spectral patterns. These results demonstrate that the individual-level ROIs generated by HSI-FCN not only stably matched the manually annotated regions in space but also generally supported automatic extraction of full-band mean spectra. Automatic ROIs preserved spectral curve shapes relatively well, but reflectance amplitude shifts may still be introduced in some boundary-sensitive samples. Therefore, subsequent spectral modeling based on automatic ROIs should consider both spectral shape metrics and absolute error metrics.

## 4. Discussion

The primary contribution of this study lies in establishing an automated preprocessing workflow for forensic entomology HSI analysis rather than proposing a fundamentally new segmentation architecture. In this workflow, HSI-FCN serves as a practical segmentation component that generates insect body masks from pseudo-RGB images, which are subsequently back-projected to the original HSI data cubes for standardized specimen-level full-band reflectance extraction. This design addresses a practical bottleneck in current insect HSI studies, where spectral inputs for species discrimination, developmental-stage determination, and insect developmental age estimation still largely depend on manual ROI delineation and reflectance extraction [13,16,17,25]. In medical and related fields, normalization [26], denoising [27], band selection [28], background segmentation [29], and ROI selection [30] have been recognized as decisive preprocessing steps affecting the quality of downstream HSI analysis. This study therefore focused on the preprocessing stage of HSI analysis and established a complete workflow from automatic insect body segmentation and individual-level ROI generation to full-band mean spectral extraction, providing a more standardized and reproducible preprocessing strategy for forensic and related insect HSI applications.

The multi-model comparison showed that HSI-FCN outperformed LightSegCNN, U-Net, U-Net+SimCLR, and YOLOv8n-seg in terms of Dice, IoU, Precision, and Accuracy. The skip connections in U-Net help transmit shallow high-resolution information and recover boundary details, but shallow features may also carry background texture, shadows, and local noise [31,32]. Moreover, previous work has shown that skip connections are not uniformly beneficial across datasets and may require task-specific redesign rather than simple feature concatenation [33]. In tasks involving small insect bodies, pronounced posture variation, complex backgrounds, and a strong dependence on ROI purity, excessive shallow details may not necessarily benefit mean spectral extraction. This trade-off is particularly relevant to HSI-based spectral extraction because false-positive background pixels can directly contaminate the mean reflectance spectrum, whereas limited omission of marginal insect-body pixels may have a smaller effect on ROI-level mean spectra. HSI-FCN removes the long skip connections between the encoder and decoder, making the decoding process more dependent on high-level semantic features. This may reduce the leakage of background texture into predicted regions and thereby improve ROI purity.

In HSI-based spectral extraction for forensic entomology, the Precision of predicted regions has an important influence on ROI spectral quality. Although models such as U-Net and YOLOv8n-seg may achieve higher object coverage, this is often accompanied by reduced Precision [34,35]. The inclusion of background pixels may alter the mean reflectance within target ROIs and subsequently affect the stability of downstream spectral analysis and modeling [36,37]. In contrast, HSI-FCN can improve ROI purity while maintaining relatively good object coverage, thereby better meeting the requirements of downstream full-band spectral modeling for high-quality spectral inputs. However, the larval subset still showed a tendency toward boundary over-expansion. The low Precision of 0.7806 and the area ratio of 1.191 ± 0.105 for LA_LSLF-2 indicate over-segmentation relative to the manually annotated ROIs, although these annotations may not fully represent absolute larval boundaries. This limitation may be related to the curved and moist larval surface, weak boundary contrast, local shadows, and information loss during image resizing. In practical forensic scenarios, surface cleaning and imaging on a uniform-colored substrate can reduce interference from soil, putrefactive fluids, and other background materials, but boundary-related spectral contamination cannot be completely eliminated. Future studies should further improve ROI purity using higher-resolution inputs, boundary-aware strategies, and more difficult-background training samples.

These results also show that a single pixel-level segmentation metric is insufficient to fully determine the practical usability of automatic ROIs [38]. To address this issue, we constructed a three-level evaluation framework encompassing pixel-level segmentation, individual-level ROI correspondence, and spectral fidelity. The test results confirmed that all 204 insect individuals in the test set were successfully matched with their corresponding manual ROIs, demonstrating the high stability of HSI-FCN in instance-level conversion. More importantly, the full-band mean spectra extracted from automatic ROIs generally preserved the curve morphology of the spectra extracted from manual ROIs. The adult subset AD_CMH-1 further showed that Pearson *r* and *R*^2^ should be interpreted separately: Pearson *r* reflects the consistency of spectral curve trends, whereas *R*^2^ is more sensitive to absolute reflectance differences. The low *R*^2^ in AD_CMH-1, therefore, mainly indicates amplitude offset rather than complete loss of spectral shape. In adult species identification, automated ROI-derived spectra may still be useful for shape-based or properly normalized classification models, whereas raw-reflectance models should be interpreted cautiously. Future work should incorporate adult-specific training samples and confidence-weighted or normalized spectral extraction to improve adult spectral fidelity.

Several limitations should be acknowledged. First, the use of a three-band pseudo-RGB image input reduced model complexity and facilitated processing by two-dimensional convolutional neural networks [39,40]. However, this approach did not use the complete hyperspectral band information and may have omitted some spectral features. Second, the number of test images was limited, and the model requires further validation across more species, insect forms, background conditions, and imaging devices. In addition, manual ROIs were used as the reference standard, but manual annotation itself may involve boundary subjectivity and operator-related differences [41]. Future studies should further compare the effects of pseudo-RGB, multiband combinations, and complete HSI inputs on segmentation performance and spectral fidelity, expand the dataset size, and conduct validation across species, devices, and complex background conditions.

Overall, this study provides an enabling framework for improving the standardization and reproducibility of insect HSI preprocessing. By reducing dependence on manual ROI delineation, the proposed approach facilitates high-throughput spectral extraction and provides a foundation for future construction of standardized spectral databases and downstream applications in forensic entomology, agriculture, and forestry-related insect studies.

## 5. Conclusions

This study developed and validated an HSI-FCN-based workflow for automatic insect body segmentation and individual-level spectral extraction from insect hyperspectral images. Using three-band pseudo-RGB images as input, the proposed method back-projects the predicted masks onto the original HSI data cube, thereby enabling automatic insect body segmentation, individual-level ROI generation, and full-band mean spectral extraction. On the independent test set, HSI-FCN outperformed LightSegCNN, U-Net, U-Net+SimCLR, and YOLOv8n-seg in terms of Dice, IoU, Precision, and Accuracy. Individual-level evaluation showed that all 204 test individuals were successfully matched with their corresponding manual ROIs. Spectral fidelity analysis further demonstrated that the full-band mean spectra extracted from automatic ROIs generally preserved the main curve morphology of the spectra extracted from manual ROIs. These results indicate that HSI-FCN can reduce dependence on manual ROI delineation and provide a feasible automated preprocessing method for standardized spectral extraction in insect HSI analysis. Future studies should further expand the dataset and validate the generalization ability of this method across additional insect taxa, more complex backgrounds, and diverse imaging conditions.

## Figures and Tables

**Table 1:** Dataset composition and independent test images used in this study.

Subset	Species	Sample Type	Total Images	Total Annotated Individuals	Test Image(s)	Annotated Individuals in Test Image(s)	Effective Bands
AD	Five insect species	Adult	8	256	AD_CMH-1	32	150
LA	Two insect species	Larva	10	262	LA_LSLF-2	22	128
SF20	*Sarcophaga formosensis*	Pupa	15	450	SF20-4; SF20-11	60	150
SF25	*Sarcophaga formosensis*	Pupa	11	330	SF25-9	30	150
SF30	*Sarcophaga formosensis*	Pupa	9	270	SF30-7	30	150
SP25	*Sarcophaga peregrina*	Pupa	10	300	SP25-2	30	150
Total	—	—	63	1868	7 images	204	—

**Table 2:** Segmentation performance comparison among five representative models on the independent test set.

Model	Representative Role	Dice	IoU	Precision	Recall	Accuracy
HSI-FCN	FCN without skip connections	0.9079	0.8328	0.9012	0.9188	0.9898
LightSegCNN	Lightweight CNN	0.8997	0.8210	0.8687	0.9403	0.9886
U-Net	U-Net baseline	0.8888	0.8036	0.8233	0.9687	0.9869
U-Net+SimCLR	U-Net+SimCLR pretraining	0.8725	0.7841	0.8566	0.8960	0.9868
YOLOv8n-seg	Instance segmentation baseline	0.8720	0.7746	0.8030	0.9594	0.9847

**Table 3:** Individual-level ROI matching and geometric consistency of HSI-FCN predictions.

Test Image	GT Regions	Predicted Regions	Matched Regions	Area Ratio (Pred/GT)	Hausdorff Distance (px)	Polygon IoU
AD_CMH-1	32	32	32	0.912 ± 0.081	39.8 ± 11.8	0.777 ± 0.043
LA_LSLF-2	22	22	22	1.191 ± 0.105	39.2 ± 16.6	0.733 ± 0.042
SF20-4	30	30	30	0.941 ± 0.047	38.0 ± 10.2	0.853 ± 0.024
SF20-11	30	30	30	1.073 ± 0.070	34.0 ± 9.7	0.857 ± 0.033
SF25-9	30	30	30	1.093 ± 0.070	38.7 ± 8.4	0.854 ± 0.046
SF30-7	30	30	30	0.913 ± 0.035	37.3 ± 8.5	0.869 ± 0.021
SP25-2	30	30	30	1.063 ± 0.054	37.2 ± 8.3	0.883 ± 0.023
Overall	204	204	204	1.019 ± 0.116	37.7 ± 10.6	0.836 ± 0.059

**Table 4:** Spectral fidelity of HSI-FCN-derived ROIs compared with GT ROIs.

Test Image	*n*	Bands	SAM (°)	Pearson *r*	*R* ^2^	RMSE
AD_CMH-1	32	150	5.80 ± 2.66	0.9951 ± 0.0040	0.244 ± 0.980	0.0159 ± 0.0101
LA_LSLF-2	22	128	2.02 ± 0.79	0.9686 ± 0.0236	0.921 ± 0.072	0.0259 ± 0.0102
SF20-4	30	150	4.50 ± 1.90	0.9993 ± 0.0007	0.959 ± 0.039	0.0206 ± 0.0090
SF20-11	30	150	1.13 ± 0.98	0.9999 ± 0.0000	0.997 ± 0.007	0.0043 ± 0.0035
SF25-9	30	150	1.91 ± 1.49	0.9999 ± 0.0001	0.986 ± 0.024	0.0062 ± 0.0048
SF30-7	30	150	4.67 ± 1.83	0.9997 ± 0.0007	0.891 ± 0.088	0.0167 ± 0.0068
SP25-2	30	150	0.97 ± 0.50	0.9999 ± 0.0001	0.998 ± 0.003	0.0058 ± 0.0033
Overall	204	—	3.06 ± 2.45	0.9956 ± 0.0123	0.848 ± 0.467	0.0132 ± 0.0103

## Data Availability

The minimal dataset supporting the main findings of this study is available from the corresponding author upon reasonable request and includes processed source data for pixel-level segmentation evaluation, model comparison, individual-level ROI matching, spectral fidelity analysis, mean spectral curve visualization, representative visual panels, and relevant analysis code. The complete raw hyperspectral data cubes are not included in the minimal dataset because of their large file size and because they are part of an ongoing dataset for related future analyses. The raw hyperspectral data cubes are available from the corresponding authors upon reasonable request where appropriate.

## Supplementary Materials

The following supporting information can be downloaded at: https://www.mdpi.com/article/10.3390/insects17080757/s1, Figure S1: Mean spectra extracted from GT ROIs and HSI-FCN-derived ROIs in the remaining test images; Table S1: Pixel-level segmentation performance of HSI-FCN on each independent test image.

## References

[1] Tran M.H., Ma L., Yuan M., Fei B. (2026). Medical hyperspectral imaging: An updated review of technology advancements and biomedical applications. J. Biomed. Opt..

[2] Zhang J., Su R., Fu Q., Ren W., Heide F., Nie Y. (2022). A survey on computational spectral reconstruction methods from RGB to hyperspectral imaging. Sci. Rep..

[3] Xing F., Chen M. (2026). Advances in Hyperspectral Imaging for Nondestructive Food Quality and Safety Detection. Foods.

[4] Zhu M., Huang D., Hu X.J., Tong W.H., Han B.L., Tian J.P., Luo H.B. (2020). Application of hyperspectral technology in detection of agricultural products and food: A Review. Food Sci. Nutr..

[5] Mahlein A.K., Kuska M.T., Behmann J., Polder G., Walter A. (2018). Hyperspectral Sensors and Imaging Technologies in Phytopathology: State of the Art. Annu. Rev. Phytopathol..

[6] Berger K., Verrelst J., Féret J.B., Wang Z., Wocher M., Strathmann M., Danner M., Mauser W., Hank T. (2020). Crop nitrogen monitoring: Recent progress and principal developments in the context of imaging spectroscopy missions. Remote Sens. Environ..

[7] Jiang S., Ma D., Tan X., Yang M., Jiao Q., Xu L. (2023). Bibliometric analysis of the current status and trends on medical hyperspectral imaging. Front. Med..

[8] Lai C.L., Karmakar R., Mukundan A., Natarajan R.K., Lu S.C., Wang C.Y., Wang H.C. (2024). Advancing hyperspectral imaging and machine learning tools toward clinical adoption in tissue diagnostics: A comprehensive review. APL Bioeng..

[9] Lu G., Fei B. (2014). Medical hyperspectral imaging: A review. J. Biomed. Opt..

[10] Li Q., He X., Wang Y., Liu H., Xu D., Guo F. (2013). Review of spectral imaging technology in biomedical engineering: Achievements and challenges. J. Biomed. Opt..

[11] Pradeep A.S., Babu J., Sudaroli Sandana J., Deivalakshmi S. (2024). Innovations in forensic science: Comprehensive review of hyperspectral imaging for bodily fluid analysis. Forensic Sci. Int..

[12] Scieuzo C., Rinaldi R., De Stefano F., Di Fazio A., Falabella P. (2025). The Contribution of Molecular Biology to Forensic Entomology. Insects.

[13] Zhang X., Qu H., Zhou Z., Chen S., Ngando F.J., Yang F., Xiao J., Guo Y., Cai J., Zhang C. (2024). Age Determination of *Chrysomya megacephala* Pupae through Reflectance and Machine Learning Analysis. Insects.

[14] Zhang X., Yang F., Xiao J., Qu H., Jocelin N.F., Ren L., Guo Y. (2024). Analysis and comparison of machine learning methods for species identification utilizing ATR-FTIR spectroscopy. Spectrochim. Acta Part A Mol. Biomol. Spectrosc..

[15] Hu G., Wang H., Liu Y., Cao F., Chen Z., Zhu Z., Luo J., Li Y., Li X., Liang J. (2026). Estimating the pupal age of *Dermestes ater* (Coleoptera: Dermestidae) using Fourier transform infrared spectroscopy combined with Chemometrics. Spectrochim. Acta Part A Mol. Biomol. Spectrosc..

[16] Wu H., Xia Y., Li J., Deng Z., Tian L., Shang Y., Guo Y. (2026). Temperature-dependent development and multimodal intra-puparial age estimation of *Sarcophaga formosensis* (Diptera: Sarcophagidae). Forensic Sci. Int..

[17] Tian L., Wang J., Miao S., Zhang X., Wu H., Wang H., Yang L., Li J., Zhao Y., Shang Y. (2026). Pupal age estimation of *Dermestes maculatus* (Coleoptera: Dermestidae) using hyperspectral imaging and machine learning. Spectrochim. Acta Part A Mol. Biomol. Spectrosc..

[18] Renard F., Guedria S., Palma N., Vuillerme N. (2020). Variability and reproducibility in deep learning for medical image segmentation. Sci. Rep..

[19] Ferrari V., Calvini R., Menozzi C., Costi E., Giannetti D., Hoffermans P., Maistrello L., Ulrici A. (2025). NIR hyperspectral imaging to identify damage caused by *Halyomorpha halys* on pears: Automated identification of Regions of Interest related to punctured areas. Spectrochim. Acta Part A Mol. Biomol. Spectrosc..

[20] Li W., Wu Y., Du L., Shang X., Shi J. (2025). Hyperspectral Imaging for Foreign Matter Detection in Foods: Advances, Challenges, and Future Directions. Foods.

[21] Wang S., Li C., Wang R., Liu Z., Wang M., Tan H., Wu Y., Liu X., Sun H., Yang R. (2021). Annotation-efficient deep learning for automatic medical image segmentation. Nat. Commun..

[22] Qin G., Liu H., Zhang X., Li W., Guo Y., Guo C. (2025). Semi-Supervised Medical Hyperspectral Image Segmentation Using Adversarial Consistency Constraint Learning and Cross Indication Network. IEEE Trans. Image Process..

[23] Lei T., Zhang D., Du X., Wang X., Wan Y., Nandi A.K. (2023). Semi-Supervised Medical Image Segmentation Using Adversarial Consistency Learning and Dynamic Convolution Network. IEEE Trans. Med. Imaging.

[24] Chen W., Bruzzone L., Dang B., Gao Y., Deng Y., Yu J.G., Yuan L., Li Y. (2026). REST: Holistic Learning for End-to-End Semantic Segmentation of Whole-Scene Remote Sensing Imagery. IEEE Trans. Pattern Anal. Mach. Intell..

[25] Voss S.C., Magni P., Dadour I., Nansen C. (2017). Reflectance-based determination of age and species of blowfly puparia. Int. J. Leg. Med..

[26] Lu G., Wang D., Qin X., Halig L., Muller S., Zhang H., Chen A., Pogue B.W., Chen Z.G., Fei B. (2015). Framework for hyperspectral image processing and quantification for cancer detection during animal tumor surgery. J. Biomed. Opt..

[27] Markgraf W., Feistel P., Thiele C., Malberg H. (2018). Algorithms for mapping kidney tissue oxygenation during normothermic machine perfusion using hyperspectral imaging. Biomed. Tech..

[28] Li Y., Chen H., Li W., Yu M., Deng J., Lv Q., Liu Y., Gao S. (2025). Hyperspectral Imaging for Hepatocellular Carcinoma and Intrahepatic Cholangiocarcinoma Differentiation. J. Biophotonics.

[29] Trajanovski S., Shan C., Weijtmans P.J.C., de Koning S.G.B., Ruers T.J.M. (2021). Tongue Tumor Detection in Hyperspectral Images Using Deep Learning Semantic Segmentation. IEEE Trans. Biomed. Eng..

[30] Cui R., Yu H., Xu T., Xing X., Cao X., Yan K., Chen J. (2022). Deep Learning in Medical Hyperspectral Images: A Review. Sensors.

[31] Xu Q., Lu J., Zhang Z., Xu D., Guo C. (2026). Deep learning-based cognitive impairment brain imaging analysis: New methods, new technologies, and new paradigms. Neural Regen. Res..

[32] Song Z., Guler U., Chandrakasan A. (2026). A 23-µJ-per-frame All-on-Chip TinyML U-Net Processor for Real-Time Autonomous Image Segmentation in Miniaturized Ultrasound Devices. IEEE Trans. Biomed. Circuits Syst..

[33] Wang H., Cao P., Wang J., Zaiane O.R. (2022). UCTransNet: Rethinking the Skip Connections in U-Net from a Channel-Wise Perspective with Transformer. Proc. AAAI Conf. Artif. Intell..

[34] Azad R., Aghdam E.K., Rauland A., Jia Y., Avval A.H., Bozorgpour A., Karimijafarbigloo S., Cohen J.P., Adeli E., Merhof D. (2024). Medical Image Segmentation Review: The Success of U-Net. IEEE Trans. Pattern Anal. Mach. Intell..

[35] Hu M., Hu X., Zhao J., Zhan H. (2025). Weld Seam ROI Detection and Segmentation Method Based on Active-Passive Vision Fusion. Sensors.

[36] Vidal M., Amigo J.M. (2012). Pre-processing of hyperspectral images. Essential steps before image analysis. Chemom. Intell. Lab. Syst..

[37] Genkawa T., Ikehata A. (2025). Background Pixel Removal for Near-Infrared Hyperspectral Images Based on the Pixel-Wise Standard Deviation of Reflectance. Appl. Spectrosc..

[38] Müller D., Soto-Rey I., Kramer F. (2022). Towards a guideline for evaluation metrics in medical image segmentation. BMC Res. Notes.

[39] Morales G., Sheppard J.W., Logan R.D., Shaw J.A. (2021). Hyperspectral dimensionality reduction based on inter-band redundancy analysis and greedy spectral selection. Remote Sens..

[40] Audebert N., Le Saux B., Lefèvre S. (2019). Deep learning for classification of hyperspectral data: A comparative review. IEEE Geosci. Remote Sens. Mag..

[41] Liao Z., Hu S., Xie Y., Xia Y. (2024). Modeling annotator preference and stochastic annotation error for medical image segmentation. Med. Image Anal..

