# Supplementary material for: Automated Individual-Level ROI-to-Spectrum Extraction for Hyperspectral Analysis in Forensic Entomology
Source: Insects. 2026 Jul 23;17(8):757. doi: 10.3390/insects17080757 (PMC13513734; doi:10.3390/insects17080757)
Supplement: Supplementary file 1 [file insects-17-00757-s001.zip › insects-4417681-supplementary.pdf]

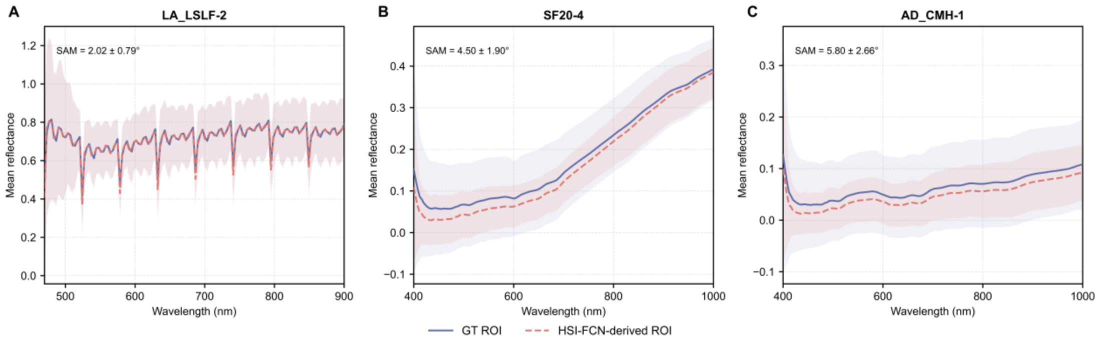

Figure S1. Mean spectra extracted from GT ROIs and HSI-FCN-derived ROIs in the remaining test images. Mean reflectance spectra were calculated from manually annotated GT ROIs and matched HSI-FCN-derived ROIs for LA_LSLF-2, SF20-4, and AD_CMH-1. Solid lines represent GT ROI spectra, and dashed lines represent spectra extracted from HSI-FCN-derived ROIs. Shaded areas indicate the standard deviation across individual ROIs. The SAM values shown in each panel summarize the spectral angular differences between the two ROI-derived spectra.

Table S1. Pixel-level segmentation performance of HSI-FCN on each independent test image.

Test image	Subset	GT individuals	Dice	IoU	Precision	Recall	Accuracy
**AD_CMH-1**	AD	32	0.8763	0.7798	0.9182	0.8380	0.9835
**LA_LSLF-2**	LA	22	0.8472	0.7350	0.7806	0.9263	0.9866
**SF20-4**	SF20	30	0.9205	0.8527	0.9500	0.8928	0.9915
**SF20-11**	SF20	30	0.9226	0.8564	0.8917	0.9557	0.9928
**SF25-9**	SF25	30	0.9204	0.8525	0.8820	0.9622	0.9912
**SF30-7**	SF30	30	0.9302	0.8695	0.9750	0.8893	0.9911
**SP25-2**	SP25	30	0.9382	0.8836	0.9110	0.9670	0.9919
**Mean**	-	**-**	**0.9079**	**0.8328**	**0.9012**	**0.9188**	**0.9898**
**SD**	**-**	**-**	**0.0332**	**0.0542**	**0.0622**	**0.0478**	**0.0034**

All metrics were calculated at the original HSI image resolution after back-projecting the predicted masks. Values in the Mean and SD rows represent the mean and standard deviation across the seven independent test images.
